# Molybdenum nanoparticle improved seed germination in Kale via regulating ROS homeostasis and metabolites accumulation under salinity stress

**DOI:** 10.3389/fpls.2026.1823191

**Published:** 2026-05-01

**Authors:** Rumeng Zhao, Lei Wang, Juanjuan Lu, Mohsin Tanveer

**Affiliations:** 1 College of Life Sciences, Xinjiang Agricultural University, Urumqi, China; 2 Key Laboratory of Ecological Safety and Sustainable Development in Arid Lands, Xinjiang Institute of Ecology and Geography, Chinese Academy of Sciences, Urumqi, China

**Keywords:** metabolite accumulation, nanoparticle, redox homeostasis, salinity, seed germination

## Abstract

Seed germination is severely constrained by soil salinity, limiting crop establishment in salt-affected agricultural lands. This study investigated whether seed priming with molybdenum nanoparticles (Mo NPs) enhances germination and early seedling growth of kale (*Brassica oleracea* var. *acephala*) under NaCl stress compared with conventional bulk molybdate treatment. Seeds were primed with 100 mg L^-^¹ Mo NPs or bulk Na_2_MoO_4_ for 12 h, then germinated under 150 mM NaCl for 3 days. Physiological markers were measured at 24 and 72 h, while untargeted metabolomics was performed on 3-day-old radicles. Seedling performance was evaluated after 10 days of 150 mM NaCl in hydroponics. Salinity reduced germination by 45% and radicle fresh weight by 49.6% compared with control. Mo NPs priming increased germination by 18.8% and radicle fresh weight by 74.8% under salinity relative to bulk Mo treatment. Mo NPs -primed seeds maintained higher GA and melatonin contents, reduced ABA accumulation, and exhibited enhanced POD activity at 24 h. Metabolomics revealed sustained starch degradation (maltose, glucose-6-phosphate), TCA cycle intermediates (malate, isocitrate), and reduced GSSG/GSH ratio in Mo NPs +NaCl compared with NaCl alone. Proline accumulation shifted from suppression at 24 h to elevation at 72 h in Mo NPs-treated seeds. Seedlings from Mo NPs-primed seeds maintained higher shoot biomass, chlorophyll content, and photosystem II efficiency under prolonged salinity. We conclude that Mo NPs priming outperforms bulk molybdate by coordinating early antioxidant defense, hormonal balance, reserve mobilization, and metabolic flexibility, enabling kale seeds to overcome the salinity-induced germination reduction.

## 1 Introduction

Soil salinization is one of the most pervasive abiotic stresses threatening global food security, with over 833 million hectares of land currently salt-affected and annual expansions of 1–2 million hectares due to poor irrigation practices and climate change (Palmgren and Shabala, 2024). In plants, salt stress triggers a cascade of physiological disruptions, including osmotic stress, ionic toxicity, and oxidative stress (Khan et al., 2020; Zhao et al., 2020). Initially, high salt concentrations reduce soil water potential, impeding water uptake and inducing physiological drought that triggers stomatal closure, reduced cell expansion, and photosynthetic inhibition (Kaur et al., 2024). Prolonged exposure leads to excessive Na^+^ and Cl^-^ accumulation, disrupting ion homeostasis and enzyme function (Flowers and Colmer, 2008; Zhao et al., 2020). Concurrently, salt stress induces oxidative stress via overproduction of reactive oxygen species (ROS), thereby further reducing plant growth (Tanveer and Ahmed, 2020; Tanveer et al., 2026). However, the salt-induced growth reduction varies across among species and developmental stages (Shahzad et al., 2022; Wang et al., 2022; Tanveer et al., 2024, 2026).

Among all developmental stages, seed germination represents the most sensitive phase in the plant life cycle under saline conditions (Chen et al., 2022; Shahzad et al., 2022; Wang et al., 2024). Successful stand establishment depends fundamentally on rapid, uniform germination, yet salt stress severely reduces germination at this initial stage (Finch-Savage and Bassel, 2016). Primarily, salt stress inhibits germination by reducing seed imbibition, altering the gibberellin/abscisic acid (GA/ABA) ratio, increasing Na^+^ toxicity during germination, and disrupting enzyme activities, particularly α-amylase (Liu et al., 2018). Third, salinity induces oxidative stress during the critical transition from quiescence to active metabolism, damaging cellular membranes and organelles before radicle protrusion can occur (Abdelgawad et al., 2016; Tlahig et al., 2021). Thus, improving seed germination and seedling establishment is essential for preparing plants to grow or survive under saline conditions.

*Brassica oleracea* var. *acephala* (kale), a leafy green vegetable of the Brassicaceae family, has gained considerable attention as a functional food due to its exceptional nutritional profile, including high concentrations of glucosinolates, flavonoids, vitamins, and minerals (Šamec et al., 2019). However, like many Brassicaceae species, kale shows moderate salinity tolerance (Pavlović et al., 2019). Studies have demonstrated that the seed germination percentage and mean emergence timing of different varieties of *B. oleracea* decrease progressively with increasing NaCl concentration, with significant reductions observed at 80 mM and near-complete inhibition at 200 mM NaCl (Zhu et al., 2011; Lema et al., 2019; Kamran et al., 2021; Hussain et al., 2023). This sensitivity at the germination stage translates directly to poor stand establishment, reduced uniformity, and ultimately lower yields in salt-affected soils (Kumar et al., 2012). Consequently, developing effective strategies to enhance kale seed germination under saline conditions is of significant agricultural importance.

Micronutrients play indispensable roles in plant adaptation to salinity stress, functioning as enzyme cofactors, osmoregulators, and components of antioxidant defense systems (Amiri et al., 2016; Aghighi Shahverdi et al., 2020; Sanoie et al., 2024). Among these, molybdenum (Mo) is essential due to its central role in nitrogen metabolism and phytohormone biosynthesis (Mendel, 2011). The Mo application increases plant growth and photosynthesis via reducing stress-induced ROS production and improves yield (Imran et al., 2021; Zulfiqar et al., 2025). In Chinese cabbage *Brassica campestris* L. ssp. *pekinensis*), Mo application has been reported to alleviate salinity-induced growth inhibition by enhancing antioxidant enzyme activities and reducing oxidative damage (Zhang et al., 2012). However, the specific effects of molybdenum on seed germination under salinity stress, particularly in kale, remain inadequately characterised.

Despite these potential benefits, conventional application of bulk molybdenum fertilizers faces significant limitations under saline conditions. Molybdate anions (MoO_4_²^-^), the primary form of Mo available to plants, compete with sulphate and phosphate for uptake, and salinity-induced ion imbalances can further reduce Mo bioavailability and acquisition by seeds (Vistoso et al., 2012; Maillard et al., 2016). In this context, nanotechnology offers transformative solutions for agricultural challenges. Nanoparticles, characterized by their ultra-small size (1–100 nm), high surface area-to-volume ratio, and unique physicochemical properties, exhibit enhanced penetration, controlled release, and targeted delivery compared to their bulk counterparts (Guo et al., 2022). Molybdenum-based nanoparticles (Mo-based NPs) have demonstrated remarkable potential in improving plant performance under various abiotic stresses (Jahangirinia et al., 2024; Li et al., 2024), however, studies specifically examining the effects of Mo NPs on seed germination under salinity stress remain scarce. Critical questions regarding how Mo NPs influence germination metabolism, reserve mobilization, osmolyte accumulation, and ROS scavenging during these vulnerable early stages of seedling establishment under saline conditions have yet to be systematically addressed. Furthermore, comparative evaluations of nano versus bulk molybdenum formulations for modulating these processes are lacking, particularly in nutritionally and economically important crops such as kale.

Therefore, the present study was designed to examine how seed priming with Mo NPs enhances kale seed germination under NaCl more effectively than bulk Mo treatment. Specifically, we aimed to: (i) compare the effects of Mo NPs and bulk Mo priming on germination parameters of kale seeds under 150 mM NaCl stress; (ii) elucidate the metabolic basis of differential germination responses through untargeted metabolomics of 3-day-old germinated seedlings; and (iii) identify critical pathways, including carbohydrate metabolism, osmolyte biosynthesis, and redox homeostasis, through which Mo NPs confer enhanced salinity tolerance during germination.

## 2 Materials and methods

### 2.1 Plant growth and experimental conditions

Seeds of *Brassica oleracea* var. *acephala* (kale) were purchased from local seed company (Beijing Dongsheng Seed Industry Co., Ltd). Prior to the experiment, seeds were surface-sterilised with 5% commercial bleach for 5–10 min, then rinsed 5 times with double-distilled water before sowing. The experiment was conducted in two independent stages: seed germination and the seedling stage. Sterilized seeds were then subjected to seed priming treatments by soaking for 12 h in either: (i) ddH_2_O (control), (ii) 100 mg L^-^¹ molybdenum nanoparticles (Mo NPs), or (iii) 100 mg L^-^¹ sodium molybdate (Na_2_MoO_4_; Mobulk) as an ionic control. Molybdenum nanoparticles (99.9% purity) were purchased from Hongwu International Group Ltd. (Huangpu, China). Following priming, seeds were gently rinsed with ddH_2_O to remove surface-adhered particles and immediately used for germination assays.

For the germination-stage assay, primed seeds (30 per petri dish) were placed on two layers of Whatman No. 43 filter paper moistened with 10 mL of either ddH_2_O (control) or 150 mM NaCl solution in 30 cm diameter petri dishes. The 150 mM NaCl concentration was selected based on preliminary experiments (**Supplementary Figure S1A**) that showed a moderate stress response without complete suppression. Petri dishes were incubated in a growth chamber at 25 ± 1 °C in darkness. Germination (radicle protrusion ≥2 mm) was recorded daily for 3 days (72h). For physiological and hormone measurment, radicles were harvested directly from these petri dishes at 24h and 72h post-stress, while untargetted metabolomics were done at 72h post-stress only. The experiment employed a completely randomized design with five petri dishes per treatment and three biological replicates per dish.

For the seedling-stage assay, primed seeds were first germinated on moist filter paper under control conditions (ddH_2_O) at 25 °C for 2 days. Uniformly germinated seedlings were then transferred to a hydroponic system containing half-strength Hoagland’s nutrient solution (pH 6.0) and allowed to acclimatise for 5 days under controlled conditions. After acclimatisation, seedlings were subjected to two treatments for 10 days: (i) control (half-strength Hoagland’s solution) and (ii) salt stress (half-strength Hoagland’s solution supplemented with 150 mM NaCl). The nutrient solution was renewed every 36h to maintain constant ion concentrations and prevent nutrient depletion.

### 2.2 Physiological measurement from germinated seeds

Several physiological stress markers in primed germinated seeds were examined after 24h and 72h post-control or NaCl incubation. To evaluate NaCl-induced oxidative stress, we measured malondialdehyde (MDA) content and hydrogen peroxide (H_2_O_2_) production. MDA levels were determined using the thiobarbituric acid (TBA) method as described by (Wu et al., 2024). Briefly, 0.1 g of radicle tissues was homogenized in a solution containing 0.5% TBA and 20% trichloroacetic acid (TCA). The mixture was incubated at 90 °C for 45 min, cooled to room temperature, and centrifuged at 15,000 rpm for 20 min. The absorbance of the supernatant was measured at 532 nm, and MDA concentration was calculated using an extinction coefficient of 155 mM^-^¹ cm^-^¹. H_2_O_2_ levels were quantified using a commercial assay kit (Beyotime, Shanghai, China) according to the manufacturer’s protocols. Later, peroxidase (POD), total soluble protein (SP), proline content, and α-amylase activity were measured using commercial assay kits (Beyotime, Shanghai, China) according to the manufacturer’s instructions. The chlorophyll content was determined using a SPAD meter (SPAD-502 Plus, Konica Minolta).

### 2.3 Untargeted metabolomics

Non-targeted metabolomic profiling was performed in radicles obtained at 3 days after germination. Frozen radicle tissues (500 mg) were homogenized with 1 mL methanol:water (3:1, v/v) for 4 min, sonicated in an ice–water bath for 5 min (three cycles), incubated at −40 °C for 1 h, and centrifuged at 17,400 *g*, 4 °C, for 15 min; supernatants were transferred to autosampler vials. Metabolites were analyzed on a Waters ACQUITY I-Class PLUS UPLC coupled to a Waters Xevo G2-XS QTof using an ACQUITY UPLC HSS T3 column (1.8 μm, 2.1 × 100 mm). In positive and negative ESI modes, mobile phase A was 0.1% (v/v) formic acid in water, and B was 0.1% (v/v) formic acid in acetonitrile; injection volume 1 μL. MS data were acquired in MSE mode (MassLynx v4.2) with alternating low/high collision energy per cycle (low CE 2 V; high CE ramp 10–40 V) at 0.2 s per spectrum. Source settings: capillary 2000 V (positive) or −1500 V (negative), cone 30 V, source 150 °C, desolvation 500 °C, cone/backflush gas 50 L h^-^¹, desolvation gas 800 L h^-^¹. Raw files were processed in Progenesis QI for peak picking/alignment and annotation against METLIN with theoretical fragment matching (mass error ≤ 100 ppm). Peak areas were normalized to the total peak area prior to analysis. PCA was used to assess within-group reproducibility and QC performance. Identified compounds were mapped to KEGG for class and pathway information. Group differences were evaluated by fold change (FC) and two-sided Student’s t-test to obtain p values. Multivariate discrimination used PLS-DA (R package ropls), with 200-time permutation testing to assess model reliability; VIP scores were obtained by cross-validation. Differential metabolites were screened using VIP > 1 and *p* < 0.05. KEGG pathway enrichment significance for differential metabolites was calculated using the hypergeometric test.

### 2.4 Statistical analysis

All experimental data are expressed as mean ± standard error (SE) of three or more independent biological replicates. Statistical significance between treatment groups was determined using one-way analysis of variance (ANOVA) followed by Tukey’s honestly significant difference (HSD) *post hoc* test, applied in Origin (2025). Differences were considered statistically significant at *p-value* < 0.05.

## 3 Results

### 3.1 Mo NPs improve seed germination under salinity stress

**Figure 1:**
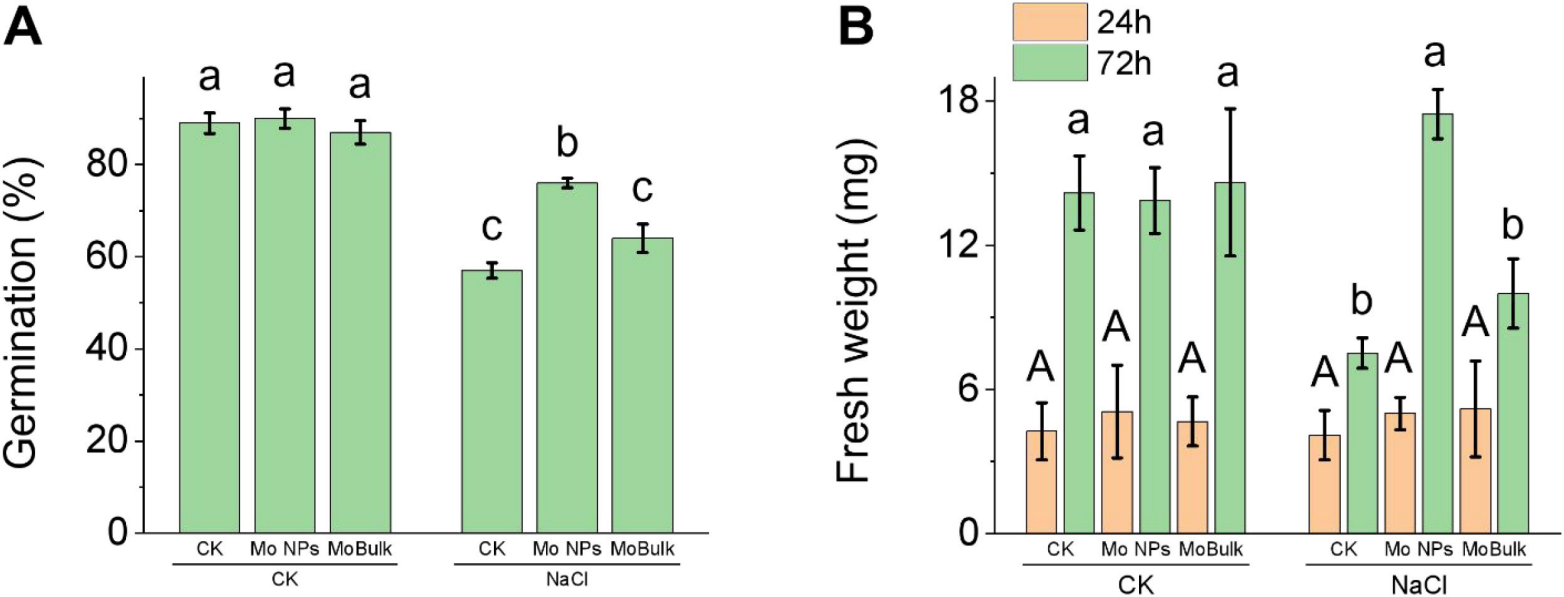
Effects of Mo NPs (molybdenum nanoparticles) and MoBulk on seed germination and seedling growth under control and salt stress conditions. **(A)** Germination percentage under control (CK) and NaCl stress treatments. Different lowercase letters indicate significant differences between treatments (P < 0.05). **(B)** Seedling fresh weight measured at 24 h and 72 h after germination under CK and NaCl conditions. Data are presented as mean ± SD. For **(A)**, different lowercase letters indicate significant differences between treatments (*p* < 0.05). For **(B)**, different uppercase letters indicate significant differences between treatments at 24 h (*p < 0.05*); different lowercase letters indicate significant differences between treatments at 72 h (*p < 0.05*).

Salinity stress severely inhibited kale seed germination by 45% compared to the control (**Figure 1A**). Further, the fresh weight of the radicle was not affected by any treatment at 24h post NaCl treatment, while significantly affected when observed at 72h post treatment (**Figure 1B**). For instance, at 72h post NaCl treatment, radicle fresh weight was reduced by 49.57% under salinity. However, Mo in either form improved seed germination and radicle fresh weight; Mo NPs outperformed MoBulk in improving these traits under salinity stress (**Figures 1A, B**). Comparing both Mo formulations, Mo NP priming increased seed germination and radicle fresh weight (at 72h) by 18.75% and 74.77%, respectively, under salinity stress compared with MoBulk priming (**Figures 1A, B**).

### 3.2 Mo NPs regulated hormone balance under salinity stress

**Figure 2:**
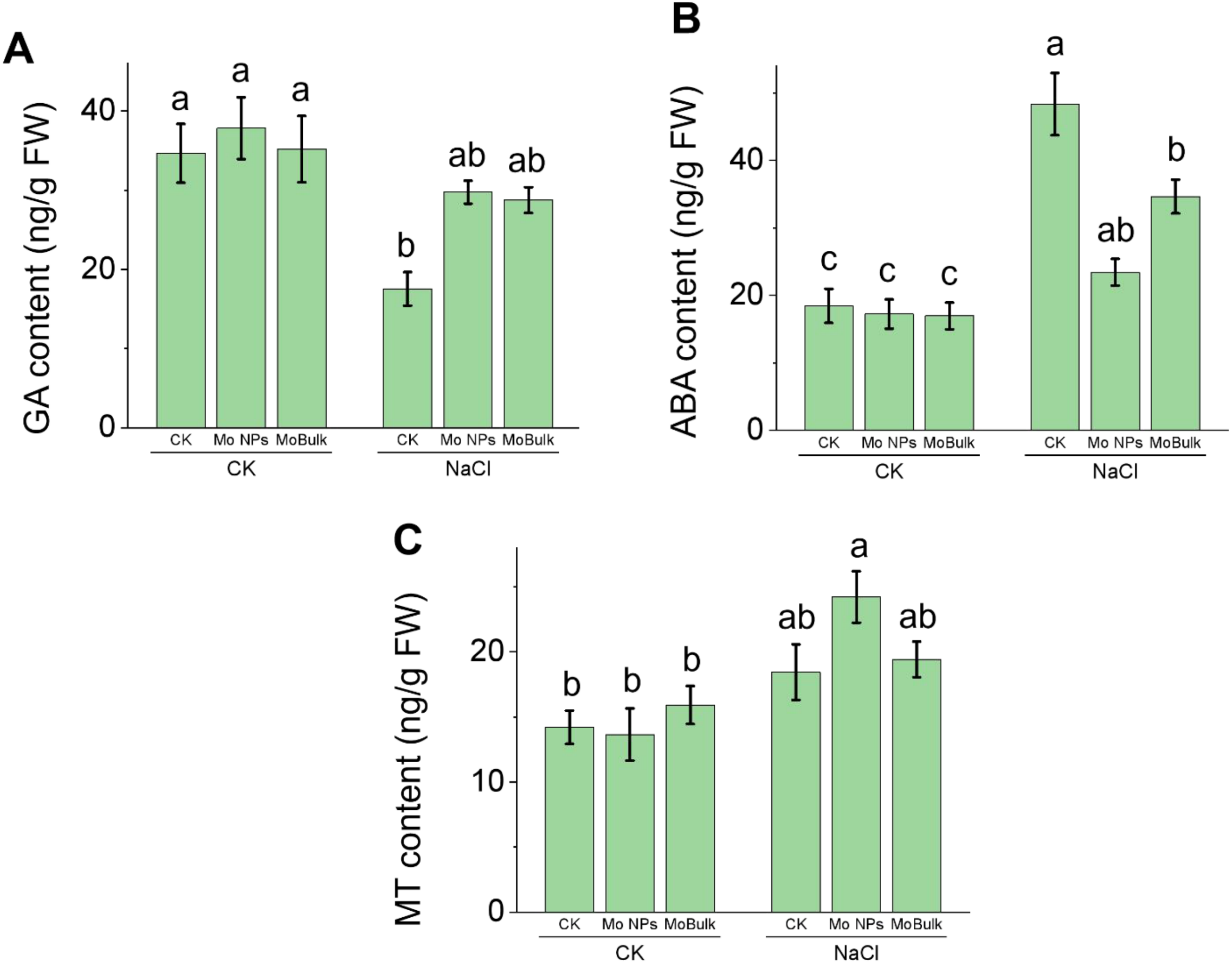
Effects of Mo NPs and MoBulk on phytohormone accumulation under control and salt stress conditions in radicles at 72 hours post-stress. **(A)** Gibberellic acid (GA) content, **(B)** abscisic acid (ABA) content, and **(C)** melatonin (MT) content in seedlings under control (CK) and NaCl stress treatments. Data are presented as mean ± SD. For each phytohormone, Different lowercase letters indicate significant differences between treatments within each condition (*p* < 0.05).

Following examination of the germination response, we also examined the contents of three important phytohormones required to regulate seed germination and radicle emergence (**Figure 2**). Salinity stress reduced gibberellin (GA) accumulation while increasing abscisic acid (ABA) accumulation. Conversely, both Mo nano formulations increased GA accumulation under salinity stress (**Figure 2A**), while Mo NPs reduced ABA accumulation by 51.53%, while MoBulk reduced ABA accumulation by 28.32% compared with alone NaCl treatment, indicating that Mo NPs regulated seed germination by regulating the GA-ABA balance more effectively than MoBulk under salinity (**Figure 2B**). Given the growth-regulatory role of melatonin (MT) in improving seed germination under stress conditions (Wang et al., 2024), we observed that MT contents increased under all treatments under salinity, with Mo NPs+salinity outperforming other treatments under salinity (**Figure 2C**). Under control conditions, no significant difference was observed in MT contents among treatments.

### 3.3 Mo NPs reduce oxidative stress in radicle under salinity stress

**Figure 3:**
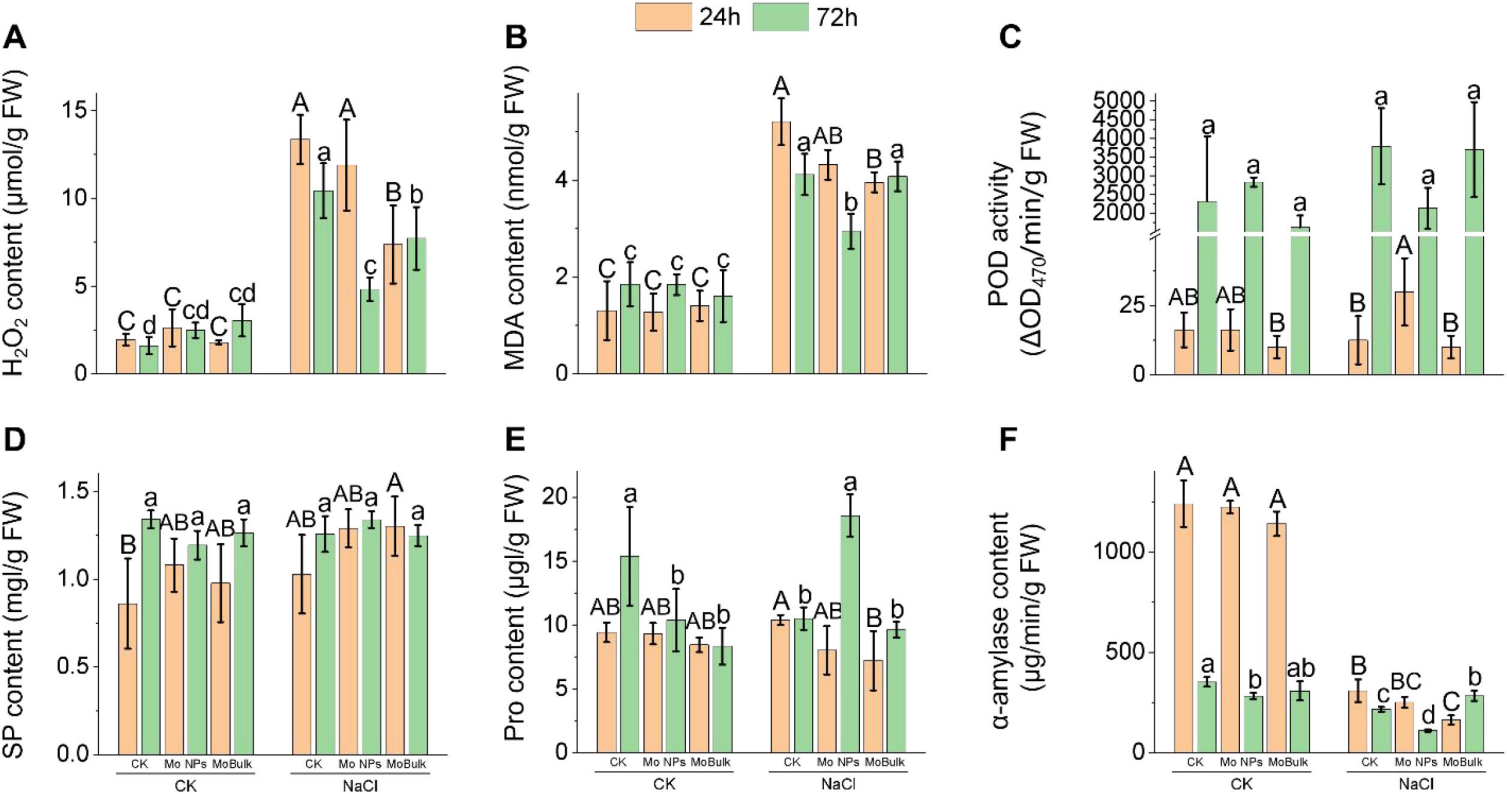
Effects of Mo NPs and MoBulk on oxidative stress markers and physiological responses under control and NaCl stress conditions. **(A)** Hydrogen peroxide (H_2_O_2_) content, **(B)** malondialdehyde (MDA) content, **(C)** peroxidase (POD) activity, **(D)** soluble protein (SP) content, **(E)** proline (Pro) content, and **(F)** α-amylase activity. Measurements were recorded at 24 h and 72 h after germination. Data are presented as mean ± SD. Different uppercase letters indicate significant differences between treatments at 24 h (*p < 0.05*); different lowercase letters indicate significant differences between treatments at 72 h (*p < 0.05*).

To further examine the radicle responses, we examined redox homeostasis in germinaing radicles at 24h and 72h post NaCl stress. Salinity significantly increased MDA content and H_2_O_2_ accumulation by 122.16% and 551.87% compared with the control (**Figures 3A, B**). However, Mo NPs priming reduced MDA contents and H_2_O_2_ accumulation by 28.46% and 53.78% respectively under salinity stress, while MoBulk priming reduced these markers only by 0.97% and 26.07% under salinity stress (**Figures 3A, B**). To reduce NaCl-induced oxidative stress, POD activity was examined as an enzymatic antioxidant in the germination-stage assay. At 24h post NaCl stress, POD activity was not affected by MoBulk priming + salinity stress and salinity treatment alone, while POD activity was increased significantly only in Mo NPs primed seeds compared with salinity (**Figure 3C**). However, at 72h post NaCl stress, POD activity was significantly higher in MoBulk priming + salinity stress and salinity treatment alone compared with Mo NPs primed seeds under salinity (**Figure 3C**).

Further, no major differences in soluble protein content were observed among treatments (**Figure 3D**), whereas proline contents showed significant differences. At 24h post-NaCl stress, proline content was higher in the salinity treatment alone than in Mo NPs/MoBulk-primed seeds. However, at 72h post NaCl, Mo NPs-primed seed showed higher proline accumulation in the radicles under salinity stress than other treatments (**Figure 3E**). Interestingly, α-amylase activity was decreased in MoBulk priming at 24 post NaCl, while at 72h post NaCl it increased (**Figure 3F**). However, Mo NPs priming showed a vice versa response under salinity stress.

### 3.4 Untargeted metabolomics in radicle under salinity stress

**Figure 4:**
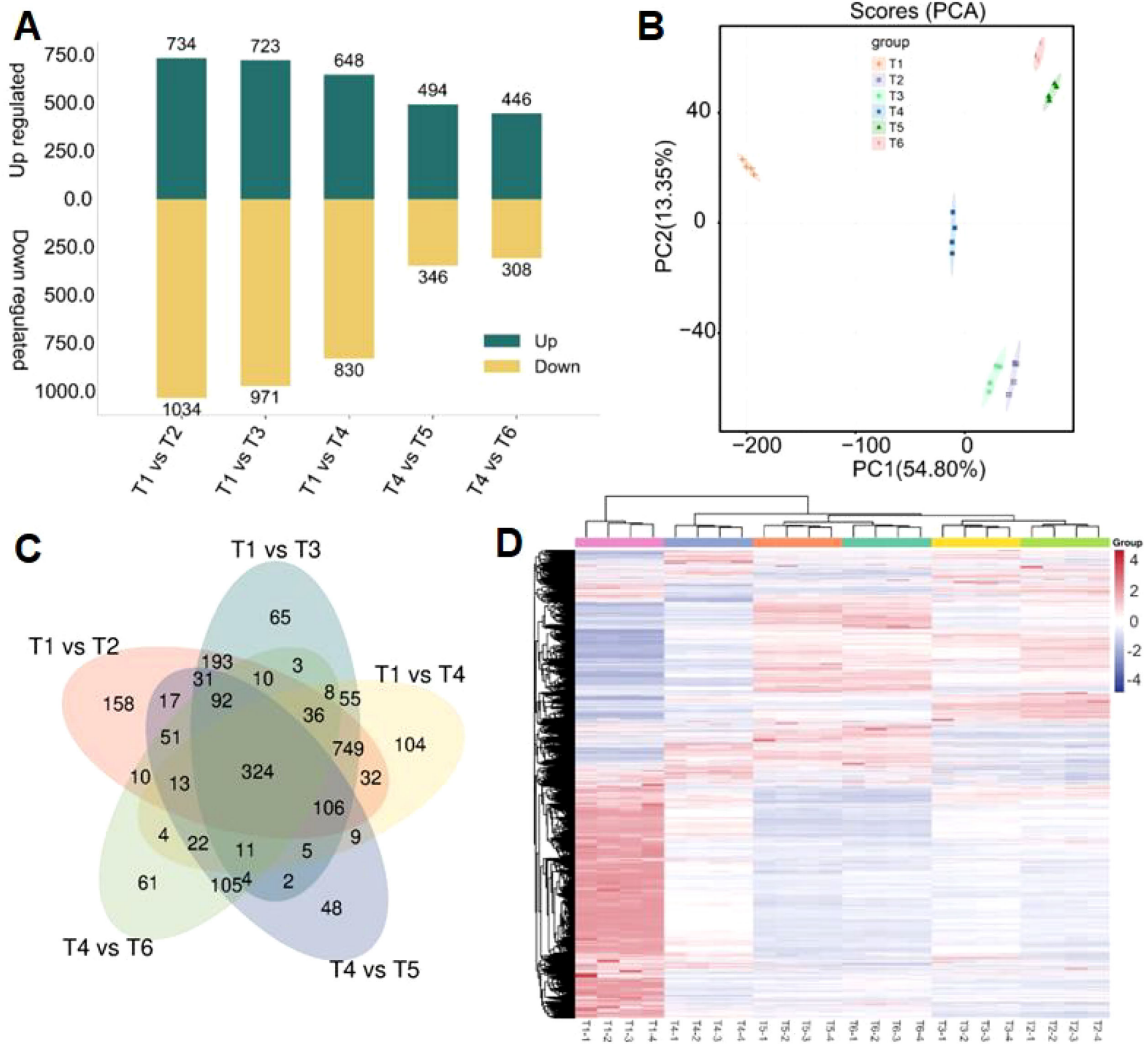
Global metabolomic reprogramming across treatments. **(A)** Number of differentially accumulated metabolites (DAMs) identified in pairwise comparisons. Treatments correspond to T1 (CK), T2 (MoBulk), T3 (Mo NPs), T4 (NaCl), T5 (MoBulk + NaCl), and T6 (Mo NPs + NaCl). **(B)** Principal component analysis (PCA) score plot showing clear separation among treatments (T1–T6). **(C)** Venn diagram illustrating shared and unique DAMs among pairwise comparisons. **(D)** Hierarchical clustering heatmap of all DAMs across treatments, showing distinct metabolic accumulation patterns among T1–T6 groups. Data represent normalized metabolite abundances.

Untargeted metabolomic profiling revealed clear metabolic reprogramming among the experimental groups (**Figure 4**; **Supplementary Table 1**). Differential analysis showed pronounced variation across comparisons (**Figure 4A**), with the largest shift in T1 vs T2 (1,768 DAMs; 734 up and 1,034 down), followed by T1 vs T3 (1,694 DAMs; 723 up and 971 down) and T1 vs T4 (1,478 DAMs; 648 up and 830 down). Fewer DAMs were detected in T4 vs T5 (840 DAMs; 494 up and 346 down) and T4 vs T6 (754 DAMs; 446 up and 308 down), indicating comparatively reduced metabolic divergence. Principal component analysis showed clear separation among treatments (**Figure 4B**), with PC1 and PC2 explaining 54.80% and 13.35% of the variance, respectively showed tight clustering of replicates and confirming data reliability. The Venn diagram identified 324 commonly regulated metabolites alongside unique sets (**Figure 4C**), while hierarchical clustering heatmap analysis further grouped samples by treatment and highlighted coordinated metabolite accumulation patterns (**Figure 4D**), supporting structured and treatment-specific metabolic shifts.

**Figure 5:**
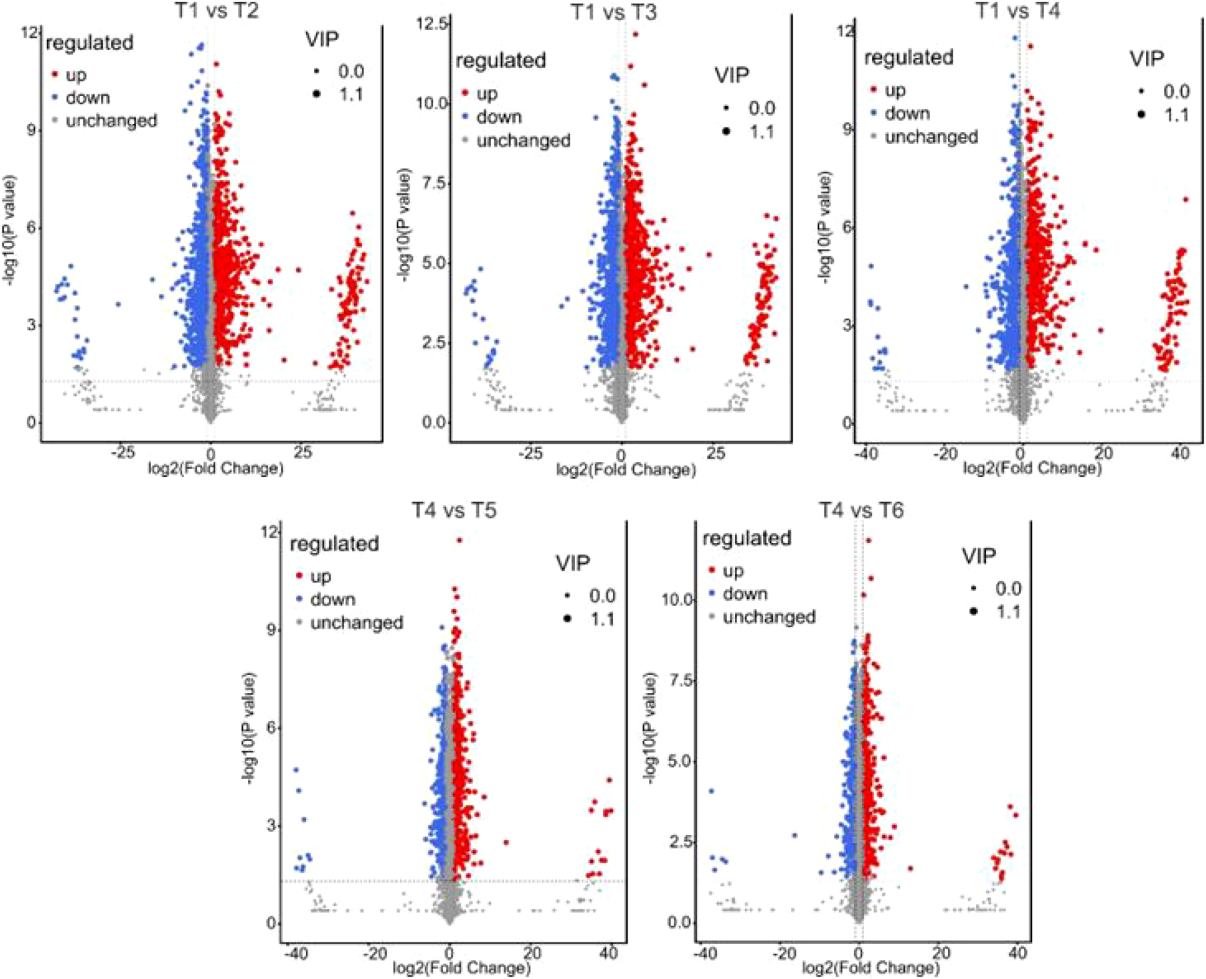
Volcano plots of differential metabolites across treatment comparisons. Volcano plots showing differentially accumulated metabolites (DAMs) for T1 vs T2, T1 vs T3, T1 vs T4, T4 vs T5, and T4 vs T6. The x-axis represents log_2_(Fold Change), and the y-axis represents −log_10_(*P* value). Red dots indicate significantly upregulated metabolites, blue dots indicate significantly downregulated metabolites, and gray dots represent unchanged metabolites. Variable importance in projection (VIP) values are indicated, and significance thresholds were applied based on fold change and statistical testing criteria. Treatments correspond to T1 (CK), T2 (MoBulk), T3 (Mo NPs), T4 (NaCl), T5 (MoBulk + NaCl), and T6 (Mo NPs + NaCl).

The Volcano plots clearly illustrated the magnitude and significance of metabolic changes across comparisons (**Figure 5**). The comparisons involving T1 (T1 vs T2, T1 vs T3, and T1 vs T4) showed a higher density of significantly upregulated and downregulated metabolites, indicating stronger metabolic shifts. In contrast, T4 vs T5 and T4 vs T6 displayed fewer significant metabolites, reflecting comparatively reduced metabolic divergence. Overall, the volcano analysis confirms that metabolic alterations were more pronounced in T1-related comparisons.

### 3.5 KEGG pathway enrichment reveals core metabolic shifts

To elucidate the functional significance of metabolic shifts associated with Mo NPs-mediated salinity tolerance, we performed KEGG pathway enrichment analysis on differentially accumulated metabolites. KEGG pathway enrichment analysis identified significant metabolic pathways across comparisons. In T1 vs T2, the major enriched pathways were D-amino acid metabolism, tryptophan metabolism, and the TCA cycle (**Supplementary Table 2**). In T1 vs T3, D-amino acid metabolism, glutathione metabolism, and phenylpropanoid biosynthesis were predominantly enriched (**Supplementary Table 3**). For T1 vs T4, alanine, aspartate and glutamate metabolism, glycolysis/gluconeogenesis, and the TCA cycle were the main pathways (**Supplementary Table 4**). In T4 vs T5 and T4 vs T6, enrichment was mainly observed in the TCA cycle, oxidative phosphorylation, and purine metabolism (**Supplementary Tables 5**, **6**).

Rather than presenting enrichment across all comparisons, we focused on three metabolic pathways that directly support the physiological outcomes observed: (i) energy metabolism and reserve mobilization, (ii) redox homeostasis and antioxidant defense, and (iii) osmotic adjustment (**Supplementary Table 7**).

Consistent with the enhanced α-amylase activity and radicle growth observed in Mo NPs-primed seeds (**Figures 1**, **3F**), pathways central to carbon and energy metabolism were prominently enriched. In Mo NPs+NaCl compared to NaCl alone (T4 vs. T6), sucrose, a key transport sugar, was elevated (log_2_FC = 0.46). TCA cycle intermediates were substantially increased, including isocitrate (log_2_FC = 1.48), aconitic acid (log_2_FC = 1.95), L-malic acid (log_2_FC = 0.62), and (S)-malic acid (log_2_FC = 1.28). In contrast, MoBulk+NaCl compared to NaCl alone (T4 vs. T5) showed smaller increases in these TCA cycle intermediates (isocitrate log_2_FC = 1.51; aconitic acid log_2_FC = 1.97; (S)-malic acid log_2_FC = 1.26), consistent with the intermediate germination phenotype of MoBulk-primed seeds.

Pathways associated with antioxidant metabolism were strongly represented in the metabolite profiles. In Mo NPs+NaCl compared to NaCl alone (T4 vs. T6), L-ascorbic acid, a key non-enzymatic antioxidant, was elevated (log_2_FC = 0.91), while oxidized glutathione (GSSG) was substantially reduced (log_2_FC = -1.65), indicating a more reduced glutathione pool. Deamino-NAD^+^, a NAD^+^ analogue involved in redox reactions, was strongly upregulated (log_2_FC = 1.72), whereas beta-NADP^+^ was downregulated (log_2_FC = -2.01). Gamma-glutamylcysteine, a precursor of glutathione biosynthesis, was elevated (log_2_FC = 0.24). These metabolites are associated with glutathione metabolism (ko00480), ascorbate and aldarate metabolism (ko00053), and nicotinate and nicotinamide metabolism (ko00760).

In MoBulk+NaCl compared to NaCl alone (T4 vs. T5), the magnitude of changes in antioxidant metabolites was reduced. L-Ascorbic acid showed modest elevation (log_2_FC = 0.43), GSSG reduction was less substantial (log_2_FC = -1.47), and gamma-glutamylcysteine showed a smaller increase (log_2_FC = 0.17), consistent with the less favorable redox status inferred from physiological measurements (**Figures 3A, B**).

For amino acid metabolism and osmotic adjustment, In Mo NPs+NaCl compared to NaCl alone (T4 vs. T6), proline, a key osmoprotectant, was elevated (log_2_FC = 1.42), consistent with physiological proline accumulation at 72 h (**Figure 3E**). Glutamine was upregulated (log_2_FC = 0.76) while glutamate was downregulated (log_2_FC = -0.75), suggesting enhanced flux through glutamine synthetase. Betaine, another compatible osmolyte, showed a modest increase (log_2_FC = 0.20). These metabolites are associated with alanine, aspartate and glutamate metabolism (ko00250) and arginine and proline metabolism (ko00330).

In MoBulk+NaCl compared to NaCl alone (T4 vs. T5), proline accumulation was intermediate (log_2_FC = 0.78), glutamine showed moderate upregulation (log_2_FC = 0.88), and betaine showed a similar modest increase (log_2_FC = 0.19), aligning with the intermediate physiological response of MoBulk-primed seeds.

### 3.6 Mo NPs priming enhances seedling growth under salinity

**Figure 6:**
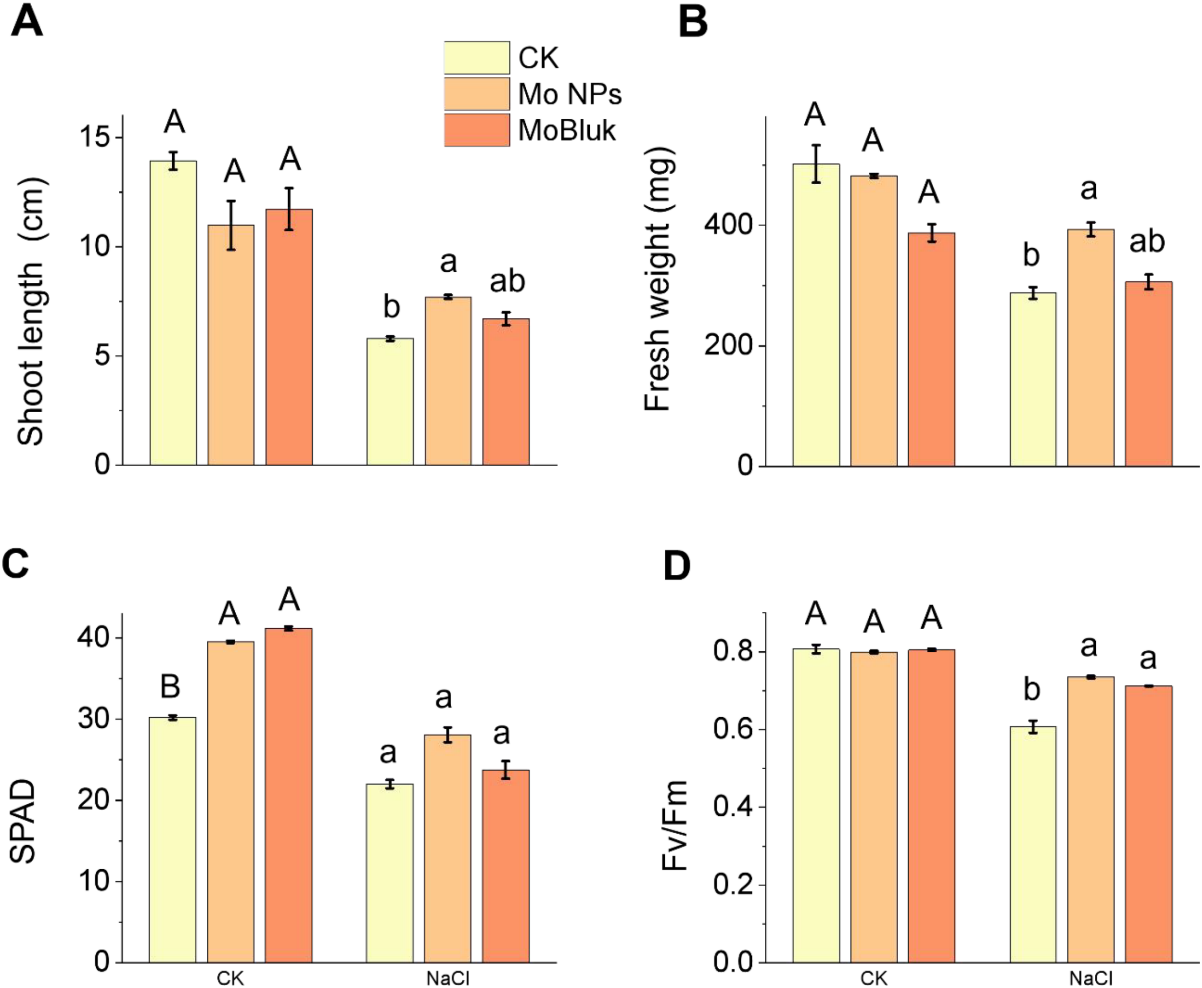
Effects of Mo NPs application on seedling growth under salinity stress. **(A)** Shoot length, **(B)** shoot fresh weight, **(C)** chlorophyll content (SPAD value), and **(D)** maximum photochemical efficiency of PSII (Fv/Fm). Mo NPs and MoBulk treated plants were subjected to control and NaCl conditions (150 mM) for 10 days and data are presented as mean ± SD. Different uppercase letters indicate significant differences between different spraying treatments under control conditions (*p* < 0.05), and different lowercase letters indicate significant differences between different spraying treatments under NaCl stress (*p* < 0.05).

**Figure 7:**
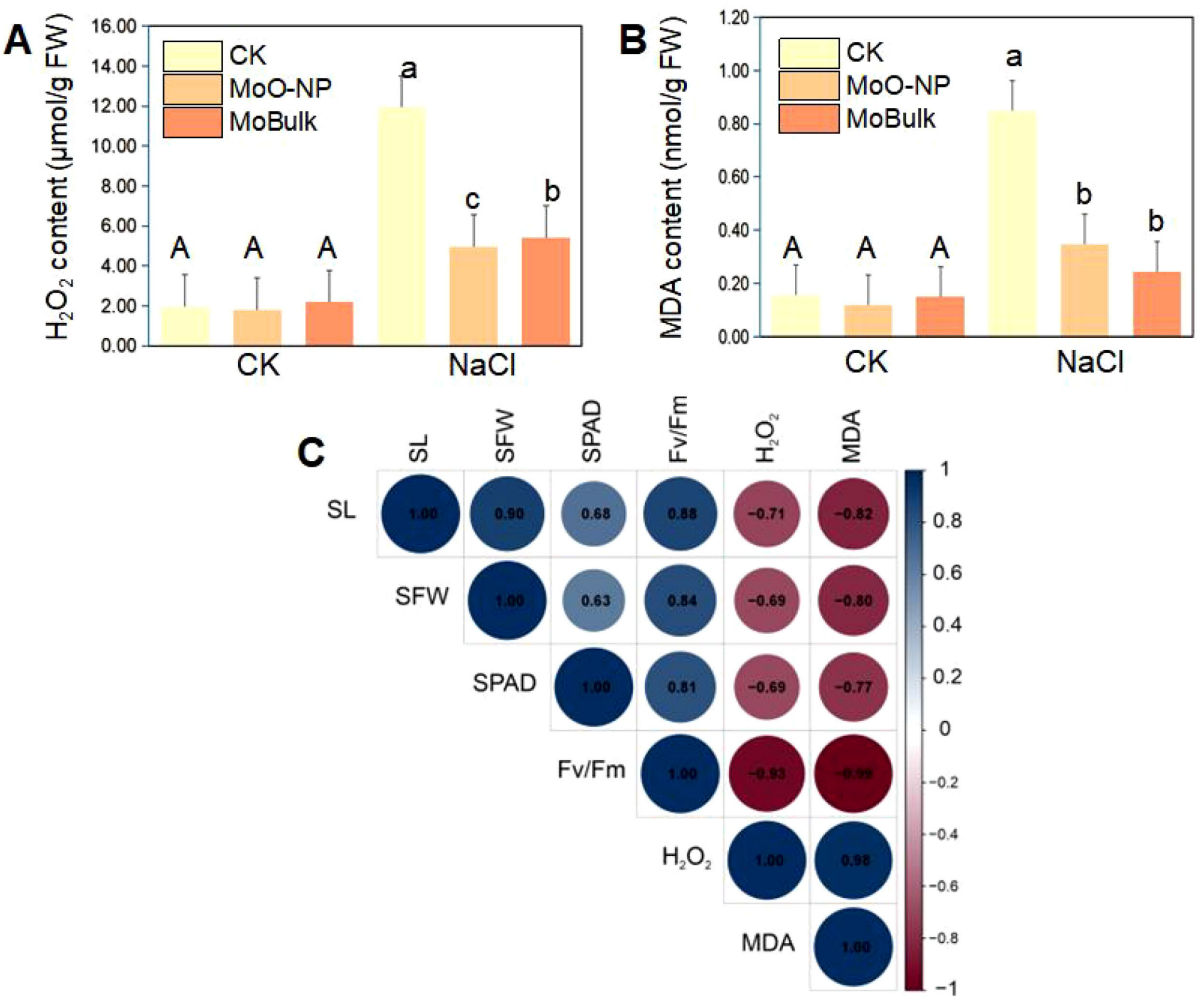
Mo NPs application alleviates oxidative damage under salinity stress and its relationship with growth traits. **(A)** Hydrogen peroxide (H_2_O_2_) content and **(B)** malondialdehyde (MDA) content in seedlings under control (CK) and NaCl conditions (150 mM). Data are expressed on a fresh weight (FW) basis and presented as mean ± SD. Different uppercase letters indicate significant differences between different spraying treatments under control conditions (*p* < 0.05), and different lowercase letters indicate significant differences between different spraying treatments under NaCl stress (*p* < 0.05). **(C)** Pearson correlation analysis among shoot length (SL), shoot fresh weight (SFW), chlorophyll content (SPAD), maximum photochemical efficiency (Fv/Fm), H_2_O_2_, and MDA. Blue circles indicate positive correlations and red circles indicate negative correlations, with color intensity and circle size proportional to correlation strength (*p < 0.05*).

After evaluating seed germination and radicle emergence in the germination-stage assay in response to nano-Mo formulations under salinity, we assessed the effects of priming in the seedling-stage assay (10 days in hydroponics under NaCl stress). The priming with Mo NPs improved seedling growth more effectively than MoBulk under salinity stress. For instance, salinity treatment (150 mM) alone reduced shoot length by 58.36% compared to the control, while Mo NPs application improved shoot length by 33.96% compared to salinity. No difference was observed between MoBulk priming + salinity and salinity stress alone (**Figure 6A**). Likewise, salinity reduced shoot fresh weight by 42.51% compared to the control, while MoBulk application improved shoot fresh weight by 6.25% under salinity. Conversely, Mo NPs application improved shoot fresh weight by 36.45% under salinity, indicating that Mo NPs application was more effective than its bulk control for improving seedling growth under salinity (**Figure 6B**). We also examined chlorophyll content (SPAD) and chlorophyll fluorescence (*Fv/Fm*). Salinity stress reduced SPAD and *Fv/Fm* significantly compared to the control, while MoBulk showed little significant effect. Conversely, the Mo NP application increased SPAD and *Fv/Fm* by 27.27% and 23.33%, respectively, compared with salinity stress alone (**Figures 6C, D**). Further, In the seedling-stage assay, salinity also increased the MDA and H_2_O_2_ contents in kale seedlings by 508% and 431.85%, respectively, compared with the control. However, Mo NPs reduced these stress marker contents by 58.30% and 58.82% under salinity, while MoBulk reduced them by 54.53% and 71.76%, respectively, under salinity (**Figures 7A, B**).

## 4 Discussion

Soil salinity severely constrains crop production worldwide (Khan et al., 2020), with seed germination representing the most vulnerable developmental bottleneck (Shahzad et al., 2022). Here, we demonstrate that Mo NPs seed priming markedly improves kale germination and early seedling establishment under NaCl stress, outperforming conventional bulk Mo treatment. Through integrated physiological and untargeted metabolomic analyses, we reveal that Mo NPs enhance salinity tolerance by (i) maintaining hormonal balance (GA/ABA) to overcome dormancy, (ii) sustaining reserve mobilisation to fuel radicle growth, (iii) reinforcing antioxidant capacity to limit oxidative damage, and (iv) remodelling primary metabolism toward energy-efficient osmotic adjustment. This multifaceted reprogramming enables Mo NPs-primed seeds to escape the salinity-induced germination blockade and establish vigorously under stress. Several supporting arguments are given below.

Since seed germination is governed by the antagonistic action of GA and ABA, with salinity stress shifting this balance towards dormancy by enhancing ABA biosynthesis and suppressing GA production (Ghosh et al., 2025). Our results confirm this antagonism, in which NaCl stress reduced GA levels while increasing ABA accumulation in germinating kale radicles (**Figures 2A, B**). Crucially, Mo NPs priming reversed this hormonal imbalance more effectively than bulk Mo treatment, increasing GA by 51.53% and reducing ABA accumulation under salinity. This differential response aligns with the physiological role of Mo as a cofactor for aldehyde oxidase (AO), a key enzyme in ABA biosynthesis (Mendel and Schwarz, 2011). We speculate that nano-formulation enables more efficient Mo delivery to embryonic tissues, fine-tuning AO activity to prevent stress-induced ABA overaccumulation while maintaining GA synthesis, a hypothesis supported by our observation of the superior performance of Mo NPs over bulk Mo despite identical elemental composition.

Notably, the MT, a pleiotropic regulator of seed germination under stress (Wang et al., 2024), increased significantly in Mo NPs-primed seeds under salinity (**Figure 2C**). MT enhances germination by upregulating GA biosynthetic genes and catabolizing ABA through the MT-ABA regulatory hub (Zhang et al., 2022). The concurrent elevation of MT along with GA and subsequence reduction in ABA in Mo NPs-treated seeds suggests that Mo NPs may activate MT-mediated hormonal crosstalk, amplifying the germination-promoting signal. This aligns with recent findings in tomato where Mo supplementation upregulated serotonin N-acetyltransferase (SNAT), a rate-limiting MT biosynthetic enzyme (Bajguz and Piotrowska-Niczyporuk, 2023). Whether Mo NPs directly enhance MT synthesis or indirectly through stress-amelioration warrants further investigation, but our data establish MT as a previously unrecognised component of Mo-mediated stress tolerance.

Given that, optimum seed germination also depends on rapid mobilization of starchy endosperm reserves to provide carbon and energy for radicle growth (Finch-Savage and Bassel, 2016). While the α-amylase is the principal enzyme hydrolysing starch, it is highly sensitive to salinity (Liu et al., 2018). Our observation that NaCl reduced α-amylase activity at 24 h post-imbibition (**Figure 3F**) is consistent with this response. Remarkably, Mo NPs-primed seeds maintained higher α-amylase activity under salinity at this critical early timepoint, while bulk Mo-primed seeds showed delayed enhancement only at 72h. This temporal advantage, early enzyme activation, likely translates directly to faster reserve mobilisation, thus explaining the 74.77% increase in radicle fresh weight in Mo NPs versus bulk Mo treatments (**Figure 1B**).

Our metabolomics data substantiate this interpretation. The consistent upregulation of maltose, glucose-6-phosphate, and glycolytic intermediates in Mo NPs+NaCl versus NaCl treatments (**Supplementary Table 7**) indicates sustained flux through starch degradation and glycolysis. Sucrose, the primary transport sugar in germinating Brassica seeds, showed treatment-specific accumulation patterns consistent with enhanced utilisation in Mo NPs-treated tissues. The sucrose/(glucose+fructose) ratio, an established index of sucrose cleavage activity (Ruan, 2014), was lower in Mo NPs+NaCl than in NaCl alone, suggesting enhanced invertase or sucrose synthase activity. This metabolic signature, elevated hexose phosphates coupled with efficient sucrose utilization, indicates that Mo NPs maintain the carbon supply line essential for radicle extension against the osmotic resistance imposed by salinity.

Salinity-induced oxidative stress is a primary cause of germination failure, with ROS overproduction damaging membranes, proteins, and DNA in the emerging radicle (Abdelgawad et al., 2016; Chakraborty et al., 2019; Manono, 2026). Our physiological data indicate a severe oxidative stress under NaCl, with MDA and H_2_O_2_ increasing 122% and 552%, respectively (**Figures 3A, B**). Mo NPs priming dramatically attenuated this oxidative burst, reducing MDA by 28.5% and H_2_O_2_ by 53.8%, whereas bulk Mo showed marginal effects at 24h. This differential protection correlates with POD activity patterns: only Mo NPs-primed seeds mounted an immediate (24h) POD response under salinity, whereas bulk Mo-primed seeds showed delayed activation at 72h (**Figure 3C**). The kinetic advantage, early, robust antioxidant deployment, is functionally significant because the initial hours of imbibition are when seeds are most vulnerable to oxidative attack (Doria et al., 2019).

Metabolomics also revealed the mechanistic basis for enhanced redox protection. The glutathione pool, central to cellular redox buffering, showed a markedly reduced GSSG/GSH ratio in Mo NPs+NaCl compared to NaCl alone (**Supplementary Table 7**), indicating maintained reducing capacity. Deamino-NAD^+^, a NAD^+^ analogue involved in redox reactions, was upregulated while beta-NADP^+^ was downregulated, consistent with enhanced NADPH generation through the pentose phosphate pathway (PPP). PPP flux is critical for germinating seeds, providing both erythrose-4-phosphate for aromatic amino acid synthesis and NADPH for antioxidant regeneration (Kruger and Von Schaewen, 2003; Scharte et al., 2023). The accumulation of ribose-5-phosphate and sedoheptulose-7-phosphate in Mo NPs-treated tissues (**Supplementary Table 7**) suggests enhanced PPP activity, linking Mo NPs treatment to improved NADPH supply for glutathione and thioredoxin systems. This aligns with the role of molybdenum in sulfite oxidase, which participates in sulfur assimilation and indirectly supports glutathione biosynthesis (HaäNsch et al., 2006; Ferri, 2017).

**Figure 8:**
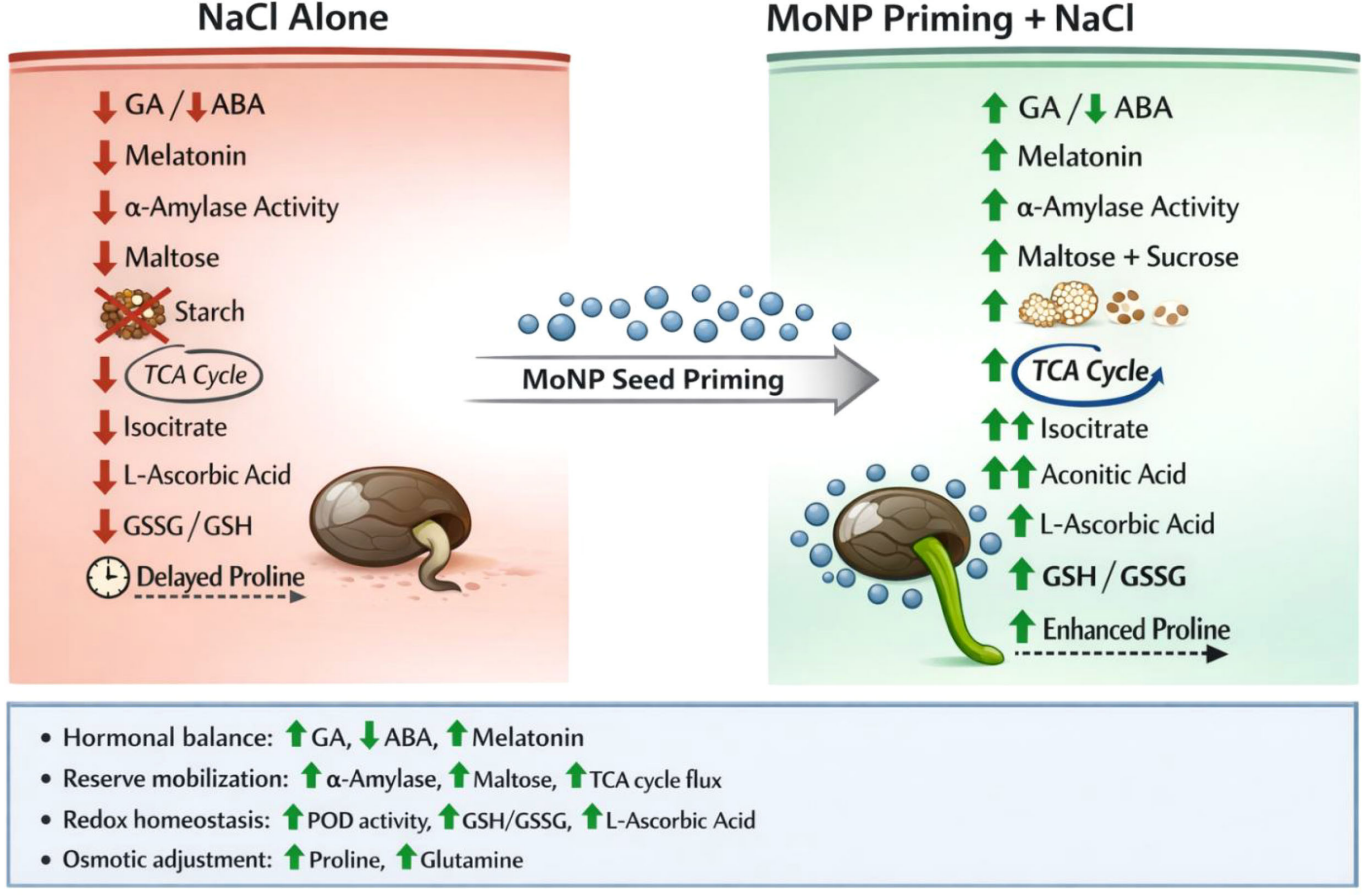
Schematic model of Mo NPs-mediated enhancement of seed germination under salinity stress. Mo NPs seed priming coordinates multiple protective mechanisms that enable kale seeds to overcome salinity-induced germination inhibition. Compared to NaCl alone (left), Mo NPs primed seeds under salinity (right) exhibit: (i) hormonal rebalancing (increased GA and melatonin; decreased ABA), (ii) sustained reserve mobilization (enhanced α-amylase activity, maltose accumulation, and TCA cycle flux), (iii) improved redox homeostasis (enhanced POD activity, reduced GSSG/GSH ratio, elevated L-ascorbic acid), and (iv) enhanced osmotic adjustment (proline and glutamine accumulation). These coordinated responses result in superior radicle growth and successful seedling establishment. Upward green coloured arrows indicate activation or increase, downwards red marked arrowsindicate inhibition or decrease. Mo NPs are depicted as blue-gray nanoparticles surrounding the primed seed.

Osmolytes accumulation also regulate seed germination (Ramazan et al., 2026). For instance, proline accumulation is a classic stress marker, but its functional interpretation requires careful temporal analysis (Forlani et al., 2019). At 24h post-NaCl, proline was highest in unstressed seeds (control and Mo NPs alone) and lowest in NaCl-treated seeds, regardless of Mo treatment (**Figure 3E**). This counterintuitive pattern, stress reducing rather than increasing proline, reflects the energy cost of proline synthesis during early germination. Proline biosynthesis from glutamate requires ATP and NADPH, resources that severely stressed seeds cannot spare when respiration is impaired (Verbruggen and Hermans, 2008). The low proline in NaCl-treated seeds at 24h thus signals metabolic paralysis, not absence of stress. By 72h, however, Mo NPs-primed seeds under salinity accumulated significantly more proline than either NaCl alone or bulk Mo+NaCl treatments (**Figure 3E**). This temporal shift, from initial suppression to later accumulation, indicates that Mo NPs-primed seeds recovered metabolic competence and could invest in osmotic adjustment once energy balance was restored. Proline at this stage functions as a compatible solute, protecting cellular structures and scavenging residual ROS (Hayat et al., 2012; Renzetti et al., 2024). The superior proline accumulation in Mo NPs-treated seeds at 72h, coupled with maintained GA levels and antioxidant capacity, indicates that Mo NPs priming enabled a coordinated, phased stress response: immediate antioxidant defense (POD, GSH) followed by sustained osmotic adjustment (proline). Bulk Mo-primed seeds, with delayed antioxidant activation, never achieved this metabolic coordination, explaining their inferior germination phenotype. Further, the accumulation of TCA cycle intermediates provides further evidence for enhanced metabolic activity in Mo NPs-primed seeds under salinity. Isocitrate, aconitic acid, and malate were significantly elevated in Mo NPs+NaCl compared to NaCl alone (log_2_FC = 1.48, 1.95, and 1.28, respectively; **Supplementary Table 7**), indicating sustained flux through the TCA cycle. This is functionally significant because germinating seeds require substantial ATP and carbon skeletons to support radicle growth against the osmotic and ionic challenges imposed by salinity. The concurrent upregulation of L-glutamine (log_2_FC = 0.76) alongside TCA intermediates suggests coordinated nitrogen assimilation, likely feeding into amino acid biosynthesis for protein synthesis in the expanding radicle. Notably, these TCA cycle intermediates were also elevated in MoBulk+NaCl but to a lesser extent (e.g., isocitrate log_2_FC = 1.51), consistent with its intermediate germination phenotype. This pattern indicates that Mo NPs priming more effectively sustains mitochondrial activity under stress, a metabolic flexibility that bulk Mo-primed seeds fail to achieve. Collectively, these results reveal that Mo NPs priming enhances salinity tolerance during germination through a coordinated network involving hormonal rebalancing, sustained reserve mobilization, improved redox homeostasis, and enhanced osmotic adjustment (**Figure 8**).

Given that, the advantages conferred by Mo NPs priming extended beyond germination to subsequent seedling growth. Mo NPs-primed plants exposed to 10 days of salinity (100 mM NaCl) maintained higher shoot biomass, chlorophyll content (SPAD), and photosystem II efficiency (Fv/Fm) than either unprimed or bulk Mo-primed plants (**Figure 6**). This carryover effect, improved performance long after the priming treatment, indicates that Mo NPs induce durable physiological changes, not merely transient protection. The sustained reduction in MDA and H_2_O_2_ in leaves of Mo NPs-primed plants (**Figure 7**) demonstrates that enhanced antioxidant capacity established during germination persists through later development. Similar priming-induced memory has been reported for other nanoparticles, with proposed mechanisms including epigenetic modifications, sustained accumulation of protective proteins, and altered hormone sensitivity (Balmer et al., 2015; Waqas et al., 2019). For Mo specifically, the persistence may reflect incorporation into molybdoenzymes (nitrate reductase, xanthine dehydrogenase) that continue functioning throughout seedling growth, maintaining nitrogen assimilation and purine catabolism under stress (Mendel and Haènsch, 2002).

An additional consideration when interpreting the inferior performance of MoBulk priming relative to Mo NPs is the chemical form of the bulk treatment. Sodium molybdate (Na_2_MoO_4_) was used as the bulk Mo source, which introduces additional Na^+^ into the priming solution. Given that salinity stress is primarily characterized by Na^+^ toxicity and osmotic imbalance, the extra sodium delivered during priming could potentially exacerbate the stress experienced by MoBulk-primed seeds during subsequent germination under NaCl. While the concentration of Na^+^ from the priming solution (approximately 1.0 mM from 100 mg L^-^¹ Na_2_MoO_4_) is substantially lower than the 150 mM NaCl used during the germination assay, the cumulative ionic load during the critical early imbibition phase cannot be entirely dismissed. Mo NPs, containing no additional sodium, avoid this potential confounding factor. This distinction highlights an additional practical advantage of nano-formulated micronutrients for seed priming under saline conditions, beyond the intrinsic benefits of nanoparticle size, surface properties, and enhanced delivery efficiency. The superior performance of Mo NPs versus bulk Mo, even after accounting for this ionic consideration, suggests that nanoformulation enhances either the efficiency of Mo incorporation into molybdoenzymes or the durability of the priming effect. Future studies examining molybdoenzyme activities and gene expression over time would clarify the mechanistic basis of this nano-enabled memory.

## 5 Conclusions

This study demonstrates that Mo NPs seed priming offers a practical, effective strategy to enhance kale germination and establishment in saline soils. The mechanistic basis, hormonal rebalancing, sustained reserve mobilization, reinforced antioxidant capacity, and metabolic flexibility, provides a integrated framework for understanding nano-enabled stress tolerance. Critically, Mo NPs outperformed conventional bulk molybdate across all parameters, supporting the agricultural promise of nanotechnology for improving crop performance under abiotic stress. Several questions remain: (i) Does Mo NPs priming affect the rhizosphere microbiome in ways that further enhance salt tolerance? (ii) Are the metabolic changes observed here accompanied by epigenetic modifications that explain the persistence of priming effects? (iii) Can Mo NPs be combined with other seed treatments (biostimulants, beneficial microbes) for additive or synergistic benefits? Addressing these questions will further refine our understanding and optimize application protocols. For practical adoption, the cost-effectiveness of Mo NPs production, optimal application rates under field conditions, and environmental fate of nanoparticles must be evaluated. Nevertheless, our results provide proof-of-concept that strategic nano-nutrition during seed priming can fundamentally reprogram germination metabolism.

## Data Availability

The raw data supporting the conclusions of this article will be made available by the authors, without undue reservation.
