# Supplementary material for: Molybdenum nanoparticle improved seed germination in Kale via regulating ROS homeostasis and metabolites accumulation under salinity stress
Source: Front Plant Sci. 2026 May 1;17:1823191. doi: 10.3389/fpls.2026.1823191 (PMC13176209; doi:10.3389/fpls.2026.1823191)
Supplement: Supplementary file 1 [file SupplementaryFile1.docx]

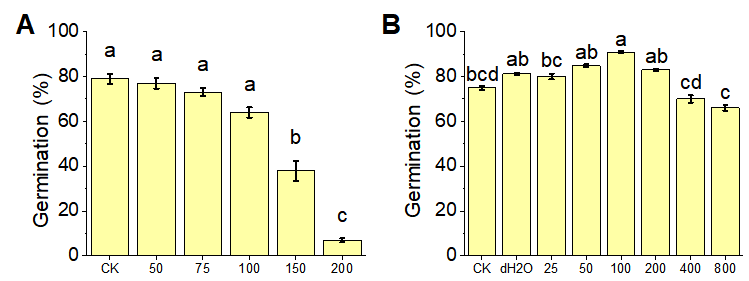


**Figure S1. Effects of NaCl and MoONPs on kale seed germination.** (A) Germination rate of kale seeds under different NaCl concentrations. (B) Germination rate of kale seeds under different concentrations of MoO-NPs. Data are presented as mean ± SD. Different lowercase letters indicate significant differences among treatments within each panel (*P* < 0.05).


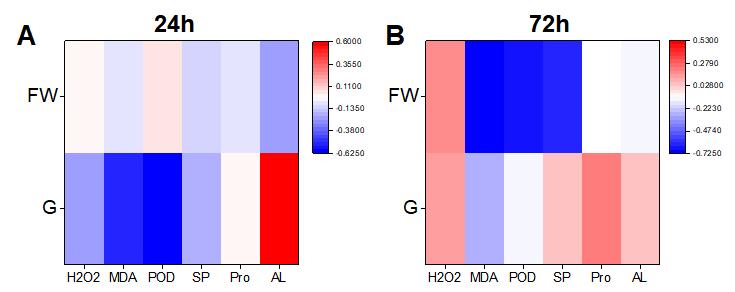


**Figure X. Relative changes in physiological and biochemical parameters under different treatments.** Heatmaps showing normalized values (Z-score transformation) of measured traits across treatments. Color gradients represent relative levels, where red indicates higher accumulation and blue indicates lower accumulation compared with the mean. (A) responses under control conditions, (B) responses under NaCl stress. Each column corresponds to a treatment, and each row represents a measured parameter.
